# Missing links in nanomaterials research impacting productivity and perceptions

**DOI:** 10.3762/bjnano.16.149

**Published:** 2025-12-03

**Authors:** Santosh Kumar Tiwari, Nannan Wang

**Affiliations:** 1 Centre for New Materials and Surface Engineering, Department of Chemistry, NMAM Institute of Technology (NMAMIT), Nitte (Deemed to be University), Nitte 574110, Karnataka, India; 2 State Key Laboratory of Featured Metal Materials and Life-cycle Safety for Composite Structures, School of Resources, Environment and Materials, Guangxi University, Nanning, Guangxi 530004, China

**Keywords:** commercialization, nanomaterials, public-facing technologies, standardization, translational barriers

## Abstract

Herein we point out critical yet often overlooked barriers restraining the real-world impact and commercial viability of nanomaterials research. In spite of decades of scientific progress, nanotechnology remains underutilized in public-facing applications. A major issue is the limited engagement of leading tech industries in developing nanotechnology-based products, prompting concerns about tangible societal and industrial outcomes. Far away, funding challenges, the field is hindered by fragmented regulations, ambiguous safety guidelines designed for bulk materials, and the absence of globally harmonized standards. These systemic limitations, coupled with persistent misconceptions, have stalled translation from lab to market. In contrast to numerous productive technologies like generative AI, machine learning, and related progress, nanotechnology has not achieved autonomous societal integration. The author argues that without a unified, transparent, and science-driven global regulatory framework, the transformative potential of nanotechnology will remain unrealized, despite over decades of excellent discoveries. This perspective calls for carefully considerations linked to productivity perception, true funding utility, and foundational reform to unlock nanotechnology’s full promise across sectors.

## Perspective

### Atom-by-atom innovation

The historical creation of nanomaterials and their applications is much older than often assumed and has long been a subject of debate. It would be wrong to believe that nanomaterials have been utilized only within the last century. In fact, several remarkable works and historical examples are well documented and discussed in the literature [1–3]. But the true turning point for nanoscience came in 1959, when physicist Richard Feynman delivered his seminal lecture, “There’s Plenty of Room at the Bottom”, at the annual meeting of the American Physical Society at Caltech, USA. This lecture provided a visionary perspective that inspired generations of researchers and greatly accelerated systematic investigations in producing and exploiting nanoscale materials. Although it took decades for technology to catch up with his ideas, his foresight catalyzed a profound shift in the way scientists approached the material world. What was once philosophical became increasingly feasible and eventually tangible. Since then, various aspects of nanoscience and nanotechnology have evolved, ranging from the examination of unique nanostructures, nanoscale characterization, metrology to nanoelectronics, nanophotonics, nanobiotechnology, nanomedicine, nanofabrication, and nanomanufacturing [4–5]. These new developments catalyzed the initiation of numerous new experimental and theoretical areas in different branches of science and engineering. Importantly, all of these studies are directly related to the creation of nanomaterials aimed at delivering much higher efficiency for specific applications compared to their conventional counterparts. In this way, progress in nanomaterial research has seen a remarkable acceleration after the discovery of fullerene by Kroto and co-workers in 1985 and witnessed an even more rapid surge following the discovery of graphene and the award of the Physics Nobel Prize in 2010 [6–7].

Graphene is often referred to as a “wonder material” due to its seemingly infinite potential in almost every domain of science and technology [8]. Its exceptional thermo-mechanical properties are ideal for critical sectors such as aerospace, medicine, space exploration, textiles, construction, and materials capable of operating from cryogenic temperatures up to beyond 2000 °C [8–9]. Similarly, other 2D nanomaterials, such as elemental 2D materials (e.g., borophene, phosphorene, and silicene), transition metal dichalcogenides (e.g., MoS_2_, WS_2_, and NbSe_2_), transition metal oxides (e.g., MnO_2_, Fe_2_O_3_, and Ni(OH)_2_ nanosheets), MXenes (e.g., Ti_3_C_2_, Ti_2_C, and Ta_4_C_3_), 2D halides (e.g., CrI_3_, NiI_2_, and FeCl_2_), 2D nitrides (e.g., BCN), 2D transition metal nitrides(e.g., MoN, Ti_4_N_3_, and GaN) [10], 2D carbides (e.g., α-C_2_N and B_2_C), 2D perovskites (e.g., (BA)_2_PbI_4_), and 2D metal-organic frameworks have also shown extraordinary promise. These nanosystems exhibit exceptional physical and chemical properties, with electronic and quantum mechanical behaviors that continue to surprise the scientific community [11]. Researchers are continuously developing and reporting new nanomaterials with unique properties tailored for diverse applications. Thus, the properties of materials once considered impossible are now accessible for real-world technological applications. In addition, nanoscience has initiated several new areas in fundamental physics and molecular dynamics, representing a profound scientific achievement of the 21st century [12]. It is noteworthy that these developments have strongly reinforced extensive collaborative and multidisciplinary research efforts around the world. Thus, at present, chemists, physicists, biologists, engineers, and computer scientists are working more closely together than ever before to design, synthesize, and apply nanosystems. Authors consider this to be one of the most important achievements in the history of science. The influence of nanomaterials research goes beyond academic curiosity. It has triggered the evolution of microscopic and analytical techniques to visualize matter at unprecedented spatial and temporal resolution, to the point where we can now visualize materials at molecular and atomic scales with unprecedented resolution. Without the demands of nanomaterials research, such advancements in instrumentation and methodology would likely have taken much longer to emerge. Notably, nanomaterial research not only has expanded the technological frontier, but has also reshaped our philosophical understanding of materials. It challenges human cognition to imagine the real-world implications of manipulating matter atom by atom. What was once the domain of speculative science fiction materials that self-heal, adapt, respond, or compute is now increasingly feasible. The boundaries between the natural and artificial, between living and nonliving systems, are being redefined through materials engineered at the nanoscale. It is important to elucidate that authors do not intend a direct comparison between nanotechnology and the fields such as generative AI, machine learning, and related progress. The latter areas are inherently multidisciplinary, encompassing multiple research domains, with nanotechnology often representing only a component. Nevertheless, from a user‑oriented perspective, generative AI, machine learning, and Internet of Things are experiencing rapid growth and receiving substantial funding with relatively few obstacles. In contrast, nanotechnology and nanomaterials research remains largely fundamental in nature, which contributes to comparatively lower levels of funding. Therefore, it would be incorrect to assume that nanotechnology has received extensive financial support and efforts but yielded limited outputs. In reality, this perception persists primarily within academic and industrial circles due to the fundamental and exploratory character of nanoscience and nanotechnology.

### Commercial adoption and mass usage remain uncertain

As discussed above, during the last three decades, extensive work has been done in various domains of nanoscience and nanotechnology, mainly focusing on large-scale production and potential consumer applications. It is estimated that, globally, investments in nanomaterials and broader nanotechnology over this period have ranged from half a trillion to nearly one trillion US dollars, roughly $400 billion from government sources and a comparable amount from industry [13–15]. Moreover, on the basis of data indexed in Web of Science and Scopus, it is evident that carbon nanotubes, graphene, metal oxides, quantum dots, and MXenes are among the most extensively studied and explored nanomaterials. These materials have been researched in nearly every discipline, leading to the establishment of numerous startups and companies focused on their production, processing, and innovative applications (Table 1). Nevertheless, in the face of such massive research efforts and innovation, widespread application and commercialization of these advanced materials remain limited [16]. The use of nanomaterials is largely confined to selected new technologies and niche innovations, with very little penetration into everyday public life. This stark gap highlights a sobering reality: Even after decades of research and good funding, the real-world integration and due consumption of nanomaterials are still minimal. When we compare the return on investment and impact with other technological revolutions such as generative AI, machine learning, Internet of Things, and related progress, nanotechnology falls short in terms of commercial and practical outcomes. It is understandable that nanotechnology is still an emerging field that is inherently more exploratory in nature. In other words, though nanomaterials research has made impressive scientific advances, its translation into mass-market products remains partial. Therefore, it raises a vital and valid question: Why are heavily researched nanomaterials such as carbon nanotubes, graphene, metal oxides, quantum dots, and MXenes still so underutilized? Surprisingly, many of us working on nanomaterials often believe that materials such as graphene, quantum dots, and MXenes are widely used in various engineering and domestic products; however, this is a misconception. The contribution of these new materials, even to cutting-edge technologies, currently is very little in commercial products. Over the past 14 years of working on these new materials, collaborating internationally, engaging with admired scientists and professors, and now leading my own research group, Santosh K. Tiwari identified a number of key reasons for underproductivity in nanotechnology. Of course, it is the author’s personal observation, but they are grounded in data and real-world experience, mostly through discussions with industry partners.

First, if we carefully examine the educational backgrounds and training of academics, scientists, engineers, and research personnel working extensively in various aspects of nanotechnology, we find that the majority come from chemistry, physics, materials science, and metallurgy. Very few come from other fields such as mechanical, energy, electronics, biomedical engineering, and mathematics. The experts belonging to chemistry, physics, and metallurgy background have been exceptional at understanding and uncovering fundamental insights into nanomaterials. They have pioneered breakthroughs and developed most of the essential tools required to industrialize nanomaterial-based products. However, they have largely not focused on the commercialization of these advancements. As a result, only a few companies are currently utilizing the potential of nanomaterials by training their workforce accordingly (Table 1). To overcome this gap, we need globally structured and domain-specific educational programs in nanoengineering, starting from secondary school through to undergraduate levels. Such specialized courses remain extremely rare worldwide. Though it is true that nanotechnology-related graduate degrees are offered globally, but most are either too broad or overly interdisciplinary, which often dilutes the focus and intent of the training. Authors, believe that engineers trained specifically in nanomaterials and nanotechnology would become valuable assets in driving innovation and entrepreneurship in this field. The authors also claim these curricula not only be domain-specific, but also include modules on manufacturing, entrepreneurship, and regulatory aspects. The regulatory aspects of nanotechnology are a complex issue worldwide. Collaboration between academia and industry in the design of these programs would ensure relevance. Early education from secondary school onwards can foster interest and build a skilled workforce ready for the nanotechnology-driven future. Meanwhile, interdisciplinary researchers can continue to push the frontiers of fundamental knowledge and discovery.

**Table 1:** List of well-known global companies and startups driving nanomaterials, nanotechnology products, and devices. Though not exclusively focused on nanotechnology, these organizations remain key players transitioning from conventional bulk materials toward advanced nanoscale innovations.

Company	Origin	Known for

NVIDIA Corp	USA	NVIDIA is most famous for advancing GPUs
Intel	USA	semiconductor devices using advanced nanoscale process nodes
Applied Materials	USA	tools/services for nanoscale device fabrication (equipment)
Samsung Electronics	South Korea	nanoscale memory, quantum dots, and logic devices in consumer electronics
Nanosys	USA	quantum-dot display materials for high-end displays
Evonik	Germany	nanosilica and functional additives for coatings and energy applications
Merck KGaA	Germany	nanomaterials for electronics, life sciences reagents, and display materials
Nanoco	UK	quantum dot materials used in bioimaging and specialty displays (commercial quantum-dot products)
Umicore	Belgium	nanocatalysts and materials for energy/catalysis applications
NAWA Technologies	France	nanotube/nanocarbon products for energy storage and composites
Nanotech Industrial Solutions	Canada	nanolubricants and surface treatment products
Johnson & Johnson	USA	nanoformulation medical products and devices
Log9 Materials	India	graphene-based products including filters and energy devices (aluminium–air solutions)
Graphmatech	Sweden	graphene-enabled additives and composite products
Kastus Technologies	Ireland	photocatalytic antimicrobial coatings that work under visible light for surfaces and touchscreens
Nanjiang Group	China	carbon nanotubes, super-strong nanomaterials, cooperating with universities for industry-applicable nanotechnology products
Naxau New Material Co.	China	nanocoating solutions: wear resistance, corrosion resistance, adjusted conductivity, thermal properties, coatings for automotive, medical, electronics
Imina Technologies	Switzerland	micro-/nano-fluidic devices used in diagnostics

Second, most nanotechnology-related products are found in big tech industries (Table 1) including space, biomedicine, and agriculture, and do not produce instantaneous and dramatic impacts that a nonscientific audience can easily perceive, unlike developments in AI, virtual reality, or other highly visible innovations. This lack of immediate, observable transformation has contributed to a strong perception, especially among political leaders, policymakers, and regulatory authorities, that nanomaterials are more of a scientific hype than practical reality [17]. As a result, there is limited motivation among these groups to actively promote and implement nanotechnology advances in public domains. Instead, they tend to continue satisfying societal needs with conventional products, which appear to be "good enough" in their view. In today’s world, there are many political figures, such as the President of the US, Mr. Trump, and his administration, who dismissed climate change and CO_2_ emissions as irrelevant. Similarly, a substantial portion of leadership at various levels believes that conventional materials are already fulfilling current demands; thus, they see no need to engage with the complexities of adopting entirely new technologies/materials. These officials, even within universities/research organizations, may appreciate research output in the form of publications and patents, but when it comes to translating that research into real-world products, they often withdraw support. This is not necessarily due to a lack of interest in innovation and efficiency, but rather because they are reluctant to engage in the rigorous and often disruptive process of establishing new policies, regulations, and frameworks required to integrate new technologies into existing systems.

Last, almost all major big tech and engineering companies are now well aware of the vast potential of nanomaterials. In fact, many of them actively support and maintain internal research and development (R&D) programs focused on various aspects of nanomaterials. However, because their existing production and engineering infrastructures are heavily optimized for bulk materials, these companies are intentionally avoiding disruptive changes in instrumentation, processing, and manufacturing tools. Of course, the goal such tech companies are to continue to profit from legacy systems, especially since most of their competitors are not yet adopting nanomaterials, although these could offer considerably better performance, sustainability, longevity, and efficiency. The founders, CEOs, and CTOs of such companies are strategically positioning themselves for a future in which the adoption of nanomaterials becomes inevitable and highly competitive. Moreover, some tech giants are already introducing nanomaterial-based products, albeit very slowly, under the guise of luxury and premium branding (Table 1). This allows them to make larger profits while gradually preparing the market. For example, Apple Inc. and Samsung are well ahead in graphene research for electronic applications [18–19]. However, highly flexible smartphones and advanced graphene-based devices remain limited, exclusive, and mostly in the pipeline. Similarly, Samsung’s quantum dot-based smart TV displays are available, but in a controlled and exclusive manner. In essence, business strategy plays a significant role in deliberately delaying the wide release of exceptionally smart and efficient nanomaterial-based products. This scenario echoes historical precedents, such as electric vehicles that were functional in London and New York [20] as early as 1925. Initially, the oil industry effectively suppressed the growth and innovation of EV technology to protect its own interests.

### The origin of nanomaterials is the real culprit

Nanomaterials hold immense potential, as highlighted in the preceding discussion. Yet, it is important to note that a very significant percentage of nanomaterials with promising applications is synthesized in labs. In contrast, naturally occurring nanomaterials are very inadequate in scale and, in most cases, are not practically useful for real-world applications, especially in the context of advanced technologies. In this regard, more than 85% of nanomaterials synthesis and production processes involve the use of hazardous chemicals such as hydrogen cyanide, sulfuric acid, phosphoric acid, nitric acid, hydrochloric acid, sodium hydroxide and potassium hydroxide [21]. To produce nanomaterials, these chemicals are not used in trace amounts but in substantial quantities. During synthesis, nearly 40–50% of these chemicals result in waste, which harms ecological systems by contaminating water, air, and soil, even when strict preventive measures are in place. Moreover, the environmental pollution caused by nanomaterials after their lifecycle, along with the chemicals used in their synthesis, is much more hazardous; up to a thousand times more compared to their bulk counterparts [22]. Additionally, most nanomaterial fabrication methods are energy-intensive. Techniques such as combustion, arc deposition, solvothermal synthesis, chemical vapor deposition, mechanical milling, and wet chemical methods require high energy input and careful process control. The ultrahigh cost of equipment, especially characterization tools used for nanosystems, are unavoidable challenges.

Another critical challenge is the precision required to produce nanomaterials. The unique properties of nanomaterials arise from controlled particle size, but achieving the desired size and uniformity is extremely difficult, even with advanced techniques. Although there are various innovative methods for producing nanomaterials, very few can consistently deliver particles with the desired properties, even under controlled experimental conditions. Therefore, after production, comprehensive characterization of nanomaterials becomes a tedious and expensive task, often more so than for advanced bulk materials. It is indispensable to acknowledge that even after detailed characterization, warranting that even 90% of the nanoparticles have uniform size and identical surface characteristics is difficult. This is largely due to the inherent instability of 0D, 1D, and 2D nanomaterials [23]. That is why, in most cases, nanomaterials require a specific medium and controlled environment for stability. In the same line, due their high surface energy, large surface area, and small size, nanomaterials naturally tend to aggregate, agglomerate, and self-assemble according to their morphology. These issues cannot be fully eliminated without external stabilization and suggestively impact their performance in real-world applications. Furthermore, such an instability becomes more pronounced as the particle size decreases. For instance, quantum dots are excellent nanomaterials, but their practical application is much more challenging compared to particles greater than 20 nm in size.

Authors believe, at present, the scientific community has explored numerous types of nanomaterial, including quantum dots, and has demonstrated their potential in the lab to address technological and societal challenges. Therefore, it is now imperative to shift the focus towards developing green and sustainable synthesis pathways. More importantly, the global scientific community must establish standardized protocols to maximize the positive impact of nanomaterials. These protocols should go over short-sighted strategies like recycling and usage restrictions. Similarly, to how systematic regulations exist for human cell research or radioactive materials, nanomaterial research must also be governed by robust guidelines to prevent uncontrolled proliferation. Otherwise, excessive production of nanomaterials for academic prestige and numerous hit-and-miss experimental works without delivering meaningful benefits may expose humanity to severe health risks, including cancer and other life-threatening diseases [24]. We are fully aware that enforcing such strict rules and close monitoring in nanomaterials research in each lab globally is not easy and may hinder scientific inquiry too. However, the scientific community must reflect on this challenge and work towards developing mutual understanding. In this context, it is worth sincerely noting that direct exposure of such chemicals (nanoscale systems) to the environment is very poorly managed worldwide, with the exception of a few countries, namely the UK, Germany, Sweden, Denmark, Netherlands, and Japan. Furthermore, the interaction of nanomaterials with biological systems remains poorly understood and could potentially lead to fatal genetic mutations.

### Funding realities in nanotechnology research

Over the past decade, discussions at major scientific conferences, in scholarly commentaries, and among policymakers have increasingly questioned the tangible returns of decades of investment in nanomaterials and nanotechnology research. This skepticism is principally salient given the substantial public and private funding committed to the field. Although it is partially true that nanotechnology has not always met the high expectations set during the “Nano-Hype” era of the early 2000s, claims that the field has failed to deliver are an oversimplification that misrepresents the nature and trajectory of scientific progress. Since the launch of flagship programs like the US national nanotechnology initiative in 2000, cumulative global investment in nanotechnology R&D has exceeded $500 billion, though notably less than the often-cited (and inflated) $1 trillion figure [13–15]. This funding has primarily supported fundamental research across materials science, chemistry, physics, and bioengineering. These efforts have led to major scientific advances, mainly in nanoelectronics, nanomedicine, catalysis, and energy storage. However, many of these contributions are embedded in downstream technologies and applications that are not always explicitly marketed as “nanotechnology”. As a result, their impact is often undervalued. In contrast, investment only in generative AI has surged dramatically in recent years. As of 2024, global funding in AI including private equity, venture capital, and governmental initiatives has surpassed $1.5 trillion [25]. This comparison often serves to highlight the perceived disparity in returns between generative AI and nanotechnology. Nonetheless, such a juxtaposition overlooks critical differences in research maturity, regulatory complexity, and commercialization pathways between the two fields.

One underappreciated aspect of nanotechnology funding is the high percentage allocated toward building sophisticated research infrastructure, predominantly in emerging economies. Unlike generative AI where a large portion of funding goes directly into product development and use, nanotechnology, still in a growing and exploratory phase has required considerable investment in physical infrastructure, metrology, safety protocols, and specialized facilities. These investments are necessary prerequisites for long-term innovation but can obscure short-term output metrics. Furthermore, in many countries such as China, India, and Brazil, some share of nanotechnology-related funding has originated from corporate social responsibility (CSR) initiatives, often mandated by government policy. Although such funding adds to the nominal investment figures, it is frequently used to establish buildings and institutional branding rather than driving core scientific innovation and product development. These CSR contributions, especially when tied to tax benefits/political goodwill, rarely undergo rigorous outcome-based auditing. As a result, much of the funding appears in national and institutional reports but contributes marginally to impactful nanotechnology outputs. A key telling example can be seen in private universities and R&D centers established by almost every large business conglomerate in countries like India. These institutions often function as vehicles for CSR spending and tax optimization, rather than as centers of cutting-edge nanoscience. Indeed, they contribute to the perception of large-scale investment in the sector, the actual return in terms of disruptive technologies and globally competitive products remains limited. This misalignment between funding appearance and outcome necessitates a more nuanced evaluation of nanotechnology’s yield. True progress in the field is inherently nonlinear and requires long-term vision. Moreover, the translational pipeline from nanoscale science to commercial technology is complex, involving multiple layers of regulation, safety, interdisciplinary integration, and market readiness.

### Need of dedicated regulatory systems

Although experts and policymakers at various levels often question the output and commercial viability of nanotechnology, the critical issue of standards and regulatory frameworks is rarely discussed and often overlooked [26]. This oversight badly hinders the broader public use of nanotechnology. Without addressing this challenge, it is impossible to envision the large-scale application of nanomaterials across technological domains. Thus, a key barrier to the commercialization of nanomaterial-based products is the absence of globally harmonized standards and coherent regulatory frameworks. A few countries actively engaged in nanotechnology have introduced nation-level regulations, but these are often not recognized internationally and may even vary across jurisdictions within the same country, mainly in federal systems. This lack of uniformity complicates cross-border commercialization and creates regulatory uncertainty for developers and industries [27]. What is especially concerning is that existing regulations are frequently adapted from frameworks originally designed for bulk materials. These are inadequate for addressing the unique physicochemical properties, size-dependent behaviors, and long-term risks associated with nanoscale materials. As a result, scientifically appropriate, dedicated regulations for nanomaterials remain either minimal or entirely absent. This regulatory fragmentation is a global issue that badly hinders the worldwide utilization of nanotechnology. The situation is further exacerbated by the rapid pace of nanotechnology advancement, mostly in critical sectors such as healthcare, energy, agriculture, and electronics. Innovation in these fields has far outpaced the development of adequate safety, ethical, and trade-related regulations. Consequently, many companies either evade pursuing nanomaterial-based products or underreport their use to bypass complex regulatory hurdles and ensure smoother market entry.

To address these challenges, a dedicated global regulatory and standardization framework is urgently needed. This should include the establishment of an international body similar to IUPAC to develop universally accepted nomenclature, definitions, and metrological standards for nanomaterials. Such standardization would enhance scientific consistency, improve industrial classification and labeling, and support transparent communication across global supply chains. Moreover, the creation of a centralized international registry for commercially available nanoproducts is highly essential. This registry would catalog product categories, safety data, and approved applications, facilitating transparency, traceability, and coordinated post-market surveillance. A further step would be the formation of a global regulatory authority analogous to the international council for harmonization in pharmaceuticals. This body would oversee ethical deployment, standardized safety evaluations, and mutual regulatory recognition across borders. Embedding “safety by design” principles within this framework is also critical. This approach will encourage the consideration of safety aspects from the earliest stages of product development, fostering responsible innovation. In the same line, it would reduce duplication in safety studies, streamline approval processes, and promote equitable access to safe nanotechnologies, especially in developing regions.

### Probable solutions

As herein, we have pointed that instead of decades of nanotechnology research, translation from lab discoveries to societal and industrial impact remains partial. To overcome these barriers, authors suggest some constructive points: (1) Establish globally harmonized, science-driven regulatory frameworks for nanomaterials, including standardized toxicity assessment, environmental fate modeling, and lifecycle analysis to enable safe and scalable commercialization. (2) Strengthen industry engagement through public–private partnerships that integrate advanced nanomaterials such as graphene, MXenes, nanoporous catalysts, and quantum dots into manufacturable products, supported by co-funded pilot projects and shared IP models. (3) Implement strategic and transparent funding models that prioritize translational potential, incorporating techno-economic analyses, reproducibility metrics, and scalability considerations to accelerate technology readiness and safeguard industrial outcomes. (4) Address persistent misconceptions via evidence-based education and outreach, supported by interactive nanomaterial property databases, AI-driven predictive safety models, and real-time tracking of industrial applications to build public trust and informed policymaking. (5) Create standardized protocols and collaborative infrastructure through inter-laboratory validated methods, centralized pilot-scale manufacturing facilities, open-access repositories, shared instrumentation, and cloud-based simulation tools for reproducibility and scale-up. (6) Develop human capital by training engineers specialized in nanotechnology, while nurturing interdisciplinary research to drive fundamental discoveries. (7) Integration of artificial intelligence, machine learning, and digital twins can accelerate materials discovery, predict safety and performance, and guide scale-up by reducing trial-and-error paths. (8) Nanotechnology deployment must embed principles of sustainability and circular economy. Designing recyclable nanomaterials, minimizing energy-intensive synthesis, and developing green manufacturing processes will be game changers.

## Conclusion

In the current era, where science is increasingly driven by commercialization, the pressure to translate fundamental research into immediate technological results has become more pronounced. However, real scientific progress is inherently incremental; it builds upon prior knowledge and often unfolds gradually over time. Nanomaterials research, like any deeply fundamental scientific domain, faces intrinsic bottlenecks, both technical and conceptual, which cannot be overlooked. In spite of these challenges, the field is undergoing a phase of foundational maturation. Although some early-stage products and devices are marketed under the umbrella of “nanotechnology”, many of these merely represent the initial wave of applications. This phase is comparable to the early era of personal computing, such as the first-generation iPhones, technologically limited, yet instrumental in paving the way for transformative advances. Criticism directed at nanomaterial research often fails to account for this temporal reality. A common argument, echoed even by some experts, is that we are producing more PhDs than the market can absorb, with insufficient corresponding innovation and job creation. It is very true and very concerning. However, paradoxically, the same institutions and voices continue to train and graduate large numbers of doctoral students each year for vested interest. This contradiction reflects a broader systemic tension within academic and industrial research ecosystems. Nanomaterials research is now experiencing a moment not unlike that faced by other maturing scientific disciplines. Premature dismissal of the contribution of the field and excessive criticism would be a strategic and intellectual misstep. Certainly, some criticisms, largely regarding output, yield, and sustainability, have partial merit, but many stem from misconceptions, media distortion, and politically motivated narratives aimed at restricting funding for fundamental research. In reality, professionals working in nanoscience and nanotechnology continue to make steady, incremental contributions. These are critical to permitting future technological platforms in energy, healthcare, computing, and advanced manufacturing.

## Data Availability

Data generated and analyzed during this study is available from the corresponding author upon reasonable request.
